# A new corrective technique for adolescent idiopathic scoliosis: convex manipulation using 6.35 mm diameter pure titanium rod followed by concave fixation using 6.35 mm diameter titanium alloy

**DOI:** 10.1186/1748-7161-10-S2-S14

**Published:** 2015-02-11

**Authors:** Hidetomi Terai, Hiromitsu Toyoda, Akinobu Suzuki, Sho Dozono, Hiroyuki Yasuda, Koji Tamai, Hiroaki Nakamura

**Affiliations:** 1 Department of Orthopaedics Surgery, Osaka City University Graduate School of Medicine, Osaka 545-8585, Japan

**Keywords:** convex manipulation, different stiffness, adolescent idiopathic scoliosis (AIS)

## Abstract

**Background:**

It has been thought that corrective posterior surgery for adolescent idiopathic scoliosis (AIS) should be started on the concave side because initial convex manipulation would increase the risk of vertebral malrotation, worsening the rib hump. With the many new materials, implants, and manipulation techniques (e.g., direct vertebral rotation) now available, we hypothesized that manipulating the convex side first is no longer taboo.

**Methods:**

Our technique has two major facets. (1) Curve correction is started from the convex side with a derotation maneuver and in situ bending followed by concave rod application. (2) A 6.35 mm diameter pure titanium rod is used on the convex side and a 6.35 mm diameter titanium alloy rod on the concave side. Altogether, 52 patients were divided into two groups. Group N included 40 patients (3 male, 37 female; average age 15.9 years) of Lenke type 1 (23 patients), type 2 (2), type 3 (3), type 5 (10), type 6 (2). They were treated with a new technique using 6.35 mm diameter different-stiffness titanium rods. Group C included 12 patients (all female, average age 18.8 years) of Lenke type 1 (6 patients), type 2 (3), type 3 (1), type 5 (1), type 6 (1). They were treated with conventional methods using 5.5 mm diameter titanium alloy rods. Radiographic parameters (Cobb angle/thoracic kyphosis/correction rates) and perioperative data were retrospectively collected and analyzed.

**Results:**

Preoperative main Cobb angles (groups N/C) were 56.8°/60.0°, which had improved to 15.2°/17.1° at the latest follow-up. Thoracic kyphosis increased from 16.8° to 21.3° in group N and from 16.0° to 23.4° in group C. Correction rates were 73.2% in group N and 71.7% in group C. There were no significant differences for either parameter. Mean operating time, however, was significantly shorter in group N (364 min) than in group C (456 min).

**Conclusion:**

We developed a new corrective surgical technique for AIS using a 6.35 mm diameter pure titanium rod initially on the convex side. Correction rates in the coronal, sagittal, and axial planes were the same as those achieved with conventional methods, but the operation time was significantly shorter.

## Introduction

Adolescent Idiopathic Scoliosis (AIS) is a three-dimensional deformity that includes scoliosis, kyphosis/lordosis, and rotation. During corrective posterior surgery, it has been believed that the concave side should be manipulated prior to the convex side because initial convex manipulation would increase the risk of vertebral malrotation, resulting in a worsened rib hump. Few papers have described convex side manipulation [1-3], so we speculated whether manipulating the convex side first is still taboo with the recent development of many materials, implants, and new manipulation techniques such as direct vertebral rotation. The purpose of this study was to introduce a new corrective technique using convex manipulation initially and a 6.35 mm pure titanium rod followed by convex fixation using a 6.35 mm titanium alloy rod. We aimed at proving its efficacy compared to conventional techniques.

## Operative technique

There are two major unique facets to our technique. (1) Curve correction is always started from the convex side with a derotation maneuver and in situ bending followed by concave rod application. (2) A 6.35 mm diameter pure titanium rod is used on the convex side and a 6.35 mm diameter titanium alloy rod on the concave side.

**Figure 1:**
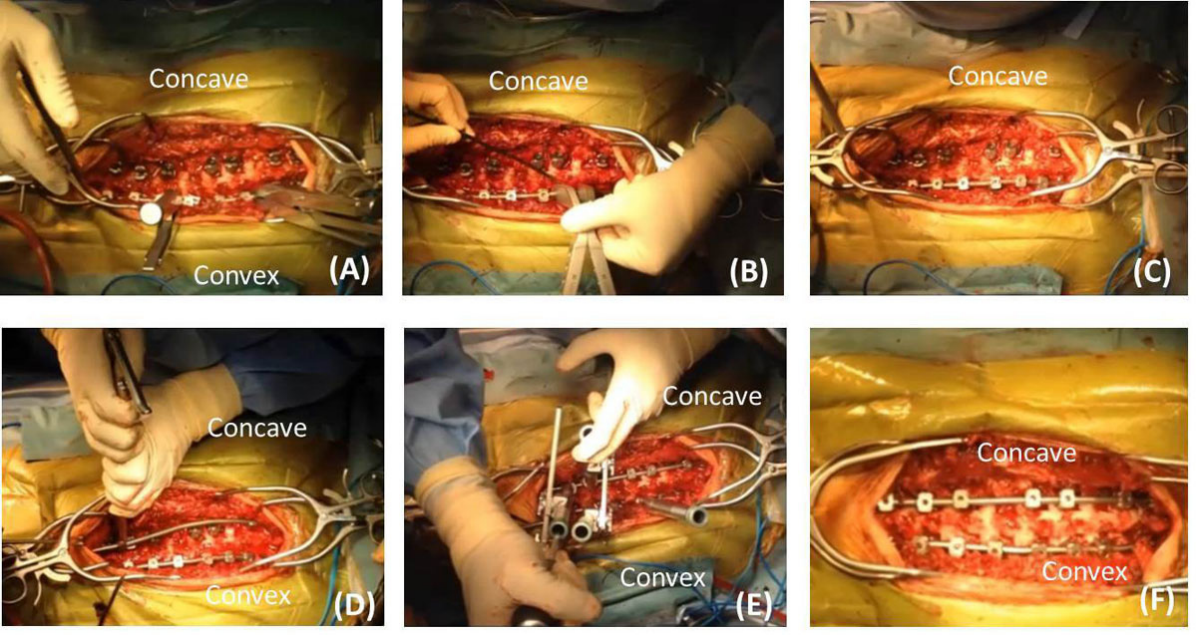
**Operative techniques.** Consecutive photographs of the technique. (A) Insertion of 6.5 mm pure titanium rod in convex side and derotation. (B) Additional adjustment with an in situ bender. (C) Curvature was almost corrected with convex manipulation. (D) The 6.5 mm titanium alloy rod was applied in the concave side. (E) Direct vertebral rotation. (F) After final adjustment with a compression/distraction maneuver.

The surgical procedure is as follows. Segmental/multi-level pedicle screws were inserted into the vertebrae of a prone patient. We preferred to use uniplaner or monoaxial screws to increase the effect of the vertebral rotation technique. A prebent 6.35 mm pure titanium rod was set in the convex side and then derotated nearly 90º by pushing the patient’s hump. After prefixed coronal and sagittal bending, we added an in situ bender under the careful observation of spinal motor evoked potentials. After the prebent 6.35 mm titanium alloy rod was introduced on the concave side, DVR was performed in the desired vertebrae, especially around the apex level. Two rods were finally fixed with two cross-links. Final adjustments were made under fluoroscopy with additional in situ bending and compression distraction. Osteotomies and bone grafting were performed in the same manner as previously described (Figure 1).

## Patients and methods

A total of 52 patients with AIS who were treated surgically from 2008 to 2012 were enrolled in this study. They were divided into two groups. Group N included 40 patients [3 male, 37 female; average age 15.9 years; Lenke type 1 (23 patients), type 2 (2), type 3 (3), type 4 (0), type 5 (10), type 6 (2)] who were treated with the new technique using 6.35 mm diameter titanium rods of different stiffness. Group C included 12 patients [all female; average age 18.8 years; Lenke type 1 (6 patients), type 2 (3), type 3 (1), type 4 (0), type 5 (1), type 6 (1)]) who were treated with conventional methods (correction started on the concave side) using 5.5 mm diameter titanium alloy rods. These data and Lenke types are detailed in Table 1. All patients had a minimum follow-up of 2 years. Radiographic parameters (Cobb angle/thoracic kyphosis/correction rates) and perioperative data were retrospectively collected and analyzed. Student’s t-test was used for statistical analysis. The present project received approval from the Ethical Commission of our institute. Written informed consent was obtained from the parents of patients for publication of this Case Report and any accompanying images. A copy of the written consent is available for review by the Editor-in-Chief of this journal.

**Table 1:** Patients sex, age, and Lenke type

Characteristic	Group N (n=40)	Group C (n=12)
Age (years)	16.0	18.8
Sex (% female)	37 (92.5%)	12 (100%)
Lenke type		
1	23 (57.5%)	6 (50%)
2	2 (5.0%)	3 (25%)
3	3 (7.5%)	1 (8.3%)
4	0 (0%)	0 (0%)
5	10 (25%)	1 (8.3%)
6	2 (5.0%)	1 (8.3%)

## Results

**Figure 2:**
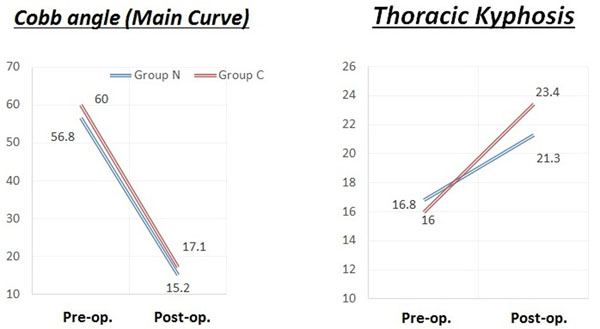
**Changes in the Cobb angle and thoracic kyphosis.***Left*: Preoperative main Cobb angles for group N/group C were 56.8°/60.0°, which improved to 15.2°/17.1° at the latest follow-up. *Right*: Thoracic kyphosis increased from 16.8° to 21.3° in group N and from 16° to 23.4° in group C.

**Figure 3:**
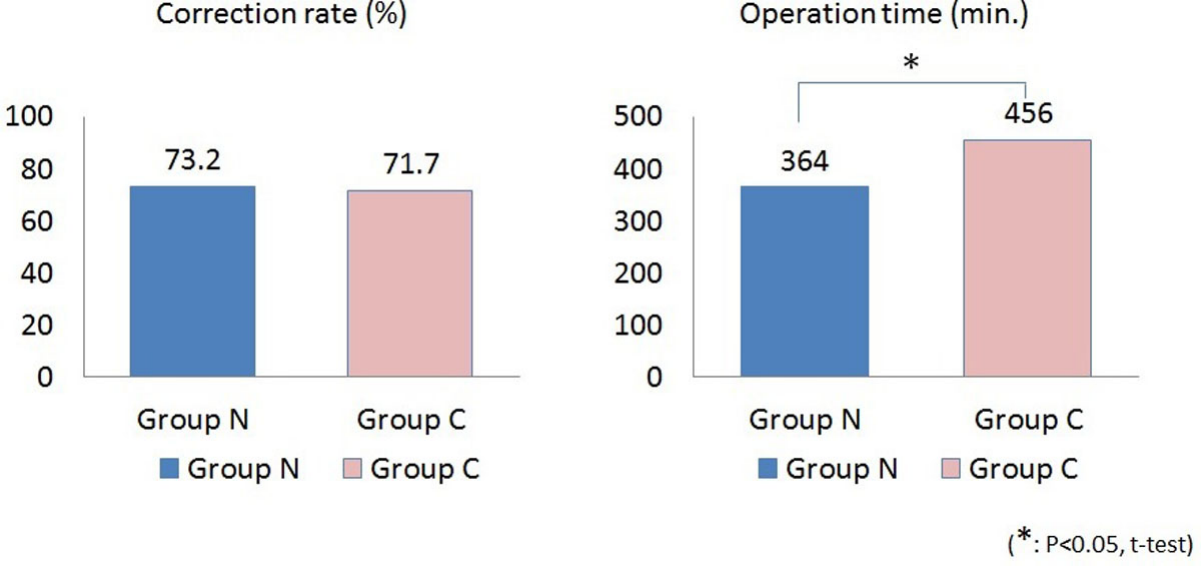
**Correction rate and Operation time.***Left*: Correction rates were 73.2% in group N and 71.7% in group C *Right*: Mean operating time in group N (364 min) was significantly shorter than that for group C (456 min).

**Figure 4:**
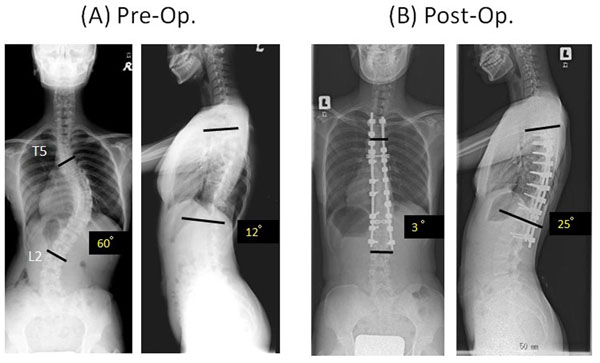
**Representative case.** A 12-year-old girl who had adolescent idiopathic scoliosis (Lenke 1AN) was treated with the new technique. (A) Preoperative anteroposterior views showed a thoracic main curve of 60° and kyphosis of 12°. (B) Postoperative views. Excellent correction was maintained at the 2-year follow-up, where the main thoracic curve is 3°, and the kyphosis is 25°.

Group N consisted of 37 females (92.5%), and group C had all females. The numbers of patients by curve type (classified by Lenke) in each group are shown in Table 1. Preoperative main Cobb angles (group N/group C) were 56.8°/60.0°, which improved to 15.2°/17.1° at the latest follow-up. Thoracic kyphosis increased from 16.8° to 21.3° in group N and from 16.0° to 23.4° in group C (Figure 2). Correction rates were 73.2% in group N and 71.7% in group C. There were no significant differences for either parameter. The mean operating time, however, was significantly shorter for group N (364 min) than for group C (456 min) (Figure 3). The apical vertebral rotation angle was investigated only in group N. It reduced from 21.9° to 17.6° in the Lenke 1, 2, and 3 curves at the latest follow-up and from 22.5° to 16.6° in the Lenke 5 and 6 curves. There were no major complications in either group. A representative case of a Lenke 1 patient is shown in Figure 4.

## Discussion

Other techniques have been reported. Ito et al. described a simultaneous double-rod rotation technique [4,5]. Chang [1,2] and Yang et al. [3] reported convex manipulation, but there are no reports on convex manipulation using rods of different stiffness. In this study, we used a 6.35 mm diameter pure titanium rod initially on the convex side. It has been believed that initial convex manipulation is not wise because of the risk of worsening the rib hump. The recent development of instruments and correction techniques, however, has allowed surgeons to manipulate the convex side before the concave side.

There are many advantages of initial convex side manipulation. First, prebent rods can be applied more easily on the convex side because the curvature is less pronounced than on the concave side. Second, the distances between the pedicle screws are greater on the convex side than those on the concave side, which is advantageous for spinal manipulation techniques such as derotation or an in situ bending maneuver because the spine can be manipulated with less load in accordance with the principles of leverage. Third, pedicle width is usually larger in the convex side than in the concave side. Therefore, pedicle screws can be inserted more easily and more safely on the convex side, which allows more screws to be inserted. Dense screws are advantageous in the manipulation process because each screw is subject to less load than are small screws placed sparsely.

We proved that the correction rates by our new technique were the same as those achieved with conventional methods that used 5.5 mm diameter titanium alloy rods with convex manipulation first. The operation time, however, was significantly shorter with the new technique.

The limitation of this study was its retrospective nature and a relatively short follow-up. Also, the new technique was used in later cases, so the surgeon’s extended experience might contribute to the bias. Further study regarding biomechanical properties and long-term follow-up is required.

## Conclusion

We developed a new corrective surgical technique using 6.35 mm diameter pure titanium rods initially on the convex side in AIS patients. The correction rates of the new method in the coronal, sagittal, and axial planes were the same as those achieved with conventional methods. The advantage of the new method is that the operation time was significantly shorter in our 52 radiographically evaluated AIS patients. Although it had been a well-known belief that concave manipulation should be started in advance of convex manipulation to avoid worsening of vertebral rotation, we proved the efficacy of initial convex side manipulation. We believe that it may be possible when only a 6.35 mm diameter pure titanium rod is used. Our method is an easy, safe technique for correcting AIS.

This is the extended abstract of IRSSD 2014 program book [6].

## Consent

Consent to publish for the article’s content and images provided as well as consent to participate were obtained from the patients and patient’s parents for this study. A copy of the written consent is available for review by the Editor-in-Chief of this journal.

## Competing interests

The author declares that he has no competing interests.

## Authors’ contributions

HT (first author) conceived the surgical modification. HTs and AS performed all the operations. KT was major contributor in writing the manuscript. SD and HY coordinated the preparation of the manuscript. HN assisted with supervision and drafting of the manuscript. All authors read and approved the final manuscript.

## Declarations

Publication charges for this collection were funded by IRSSD 2014 Sapporo.

This article has been published as part of *Scoliosis* Volume 10 Supplement 2, 2015: Research into Spinal Deformities 9: Short Papers from the IRSSD 2014 Meeting. The full contents of the supplement are available online at http://www.scoliosisjournal.com/supplements/10/S2.
